# A Genotyping Study in Benin Comparing the Carriage of *Plasmodium falciparum* Infections Before Pregnancy and in Early Pregnancy: Story of a Persistent Infection

**DOI:** 10.1093/cid/ciaa841

**Published:** 2020-06-22

**Authors:** Sayeh Jafari-Guemouri, Laura Courtois, Atika Mama, Baptiste Rouas, Gabriel Neto Braga, Manfred Accrombessi, Achille Massougbodji, Xavier C Ding, Nicaise Tuikue Ndam, Nadine Fievet, Valérie Briand

**Affiliations:** 1 Université de Paris, UMR261-MERIT, Institut de Recherche pour le Développement, Paris, France; 2 Clinical Research Institute of Benin (IRCB), Abomey-Calavi, Benin; 3 Faculty of Infectious and Tropical Diseases, Disease Control Department, London School of Hygiene and Tropical Medicine, London, United Kingdom; 4 FIND, Geneva, Switzerland; 5 University of Bordeaux, Inserm, Institut de Recherche pour le Développement, Inserm, University of Bordeaux, UMR, Bordeaux, France

**Keywords:** malaria, pregnancy, Africa, genotyping techniques, polymerase chain reaction

## Abstract

**Background:**

Malaria infections in the first trimester of pregnancy are frequent and deleterious for both mother and child health. To investigate if these early infections are newly acquired or already present in the host, we assessed whether parasites detected before pregnancy and those detected in early pregnancy are the same infection.

**Methods:**

We used data from the preconceptional “RECIPAL” study (Benin, 2014–2017). Sixty-three pregnant women of 411 included who had a malaria infection detected by quantitative polymerase chain reaction both before pregnancy and at the first antenatal care (ANC) visit were selected for this study. Two highly polymorphic markers, *msp-2* and *glurp*, and a fragment-analysis method were used to enumerate the *Plasmodium falciparum* genotypes and to quantify their proportions within isolates. An infection was considered as persistent when identical *msp-2* and *glurp* genotypes were found in the corresponding prepregnancy and early-pregnancy samples.

**Results:**

The median time between the 2 malaria screenings was 3 months. The median gestational age at the first ANC visit was 6.4 weeks. Most infections before pregnancy were submicroscopic infections. Based on both *msp-2* and *glurp* genotyping, the infection was similar before and in early pregnancy in 46% (29/63) of cases.

**Conclusions:**

Almost half of *P. falciparum* infections detected in the first trimester originate before pregnancy. Protecting young women from malaria infection before pregnancy might reduce the prevalence of malaria in early pregnancy and its related poor maternal and birth outcomes.

## Teaser

To distinguish between new and persistent malaria infection in early pregnancy, we enumerated and compared *Plasmodium falciparum* genotypes in isolates both before and in the first weeks of pregnancy. Persistent infections from preconception accounted for half the cases.

Malaria in pregnancy is a major public health problem in sub-Saharan Africa, with 39 million pregnant women at risk of *Plasmodium falciparum* infection every year [1]. Malaria has substantial risk for pregnant women (anemia), the fetus (fetal growth restriction, fetal loss), and the newborn child (low birth weight, preterm birth), leading to an increased risk of neonatal and infant mortality [2, 3]. In sub-Saharan African countries, preventive strategies against malaria associated with pregnancy are based on long-lasting insecticide–treated bed nets and intermittent preventive treatment in pregnancy (IPTp) with sulfadoxine-pyrimethamine (SP) [4]. Intermittent preventive treatment in pregnancy consists of the administration of SP at each antenatal care (ANC) visit from the second trimester of pregnancy onwards; SP is contraindicated during the first trimester because of potential teratogenic effects on the fetus. Insecticide-treated bed nets are distributed at the first ANC visit, which generally occurs around 4–5 months of pregnancy. Therefore, pregnant women remain unprotected or insufficiently protected during the first months of pregnancy, particularly during the first trimester. However, this period of fetal life may be highly critical in the case of pregnancy-associated malaria because of the sequestration of parasites and their accumulation in the placenta simultaneously with trophoblast differentiation and vascular remodelling of the uterus [2]. We recently showed that women infected in the first trimester had a significantly higher risk of maternal anemia at the end of pregnancy compared with uninfected women at the same period and that they were more likely to have low-birth-weight newborns when infected both in the first trimester and later in pregnancy [5].

Malaria is particularly frequent in the first trimester of pregnancy [6–8] and might cause up to 70% of the total exposure to placental malaria infection [9]. With data from a preconceptional cohort, we showed that women infected with malaria before pregnancy were more likely to be infected with malaria in the first trimester of pregnancy compared with uninfected women before pregnancy [6, 7]. Also, the proportion of microscopic infections was almost 2 times higher at the first ANC visit (6 weeks’ gestation) than before conception.

We therefore hypothesized that malarial infections in the first trimester of pregnancy are already present before pregnancy. Also, we assumed that most of these prepregnancy infections were submicroscopic (ie, only detectable by polymerase chain reaction [PCR]) and that most become patent and detectable with microscopy in early pregnancy. Therefore, the main objective of the present study was to investigate if parasites detected in women before pregnancy and those detected during the first weeks of pregnancy represent the same infection that persists in time.

## METHODS

### Study Population and Design

In this project we used data from a previous study, RECIPAL (REtard de Croissance Intra-utérin et PALudisme), which was conducted in the south of Benin from June 2014 to August 2017 [10]. The RECIPAL study was established to assess the effect of malaria in the first trimester of pregnancy on birth outcomes and was a preconceptional longitudinal cohort study. A total of 1214 women of reproductive age were first recruited and followed monthly until becoming pregnant. At inclusion, they were screened for malaria using both microscopy (thick blood smear test [TBS]) and quantitative PCR (qPCR). After 30 months of follow-up, 411 women became pregnant. Their pregnancy was identified with a mean of 6.9 weeks of gestation based on early ultrasound scan. Once pregnant, women were followed up monthly at the facility level for clinical examination and malaria screening (using both microscopy and qPCR). Only microscopy results were available at the time of the visit, and qPCR was postponed to the end of follow-up. Before pregnancy and from the second trimester of pregnancy onwards, women were treated with artemether-lumefantrine if infected with microscopic malaria whether or not they were symptomatic. In the first trimester of pregnancy, infected women were treated with quinine.

### Inclusion Criteria for the Genotyping Study

To eliminate the risk of new infections between the first screening before pregnancy and subsequent screening in early pregnancy, we selected, among 411 pregnant women, those with a positive PCR (proof of existence of the parasite’s DNA) both before pregnancy and at the first ANC visit during pregnancy (shortest time between the 2 infections). All pairs of PCR-positive infections, whether or not detected by microscopy, were considered. Based on these criteria, 66 women were included in this study.

### Microscopy and Quantitative Polymerase Chain Reaction Testing

Microscopy and qPCR testing were performed in the framework of RECIPAL study. Briefly, TBSs were stained with Giemsa, and parasitemia was quantified by the Lambaréné method [11]. Blood smears were considered negative if no parasites were seen in all 10-µL TBSs. The presence of *P. falciparum* was also tested in duplicate by a qPCR that targeted the 18S ribosomal DNA (rDNA) after 40 cycles of amplification [12, 13]. Starting from 50 µL of blood spot samples on filter paper, the extraction procedure leads to a final volume of 150 µL extracted DNA. A test sampling of 5 µL extracted DNA thus corresponds to 1.7 µL of blood. All parasite densities estimated for this study were determined by qPCR. Parasitemia was determined by extrapolation of cycle thresholds (Ct) from a standard curve generated with purified DNA from 3D7 *P. falciparum*-infected erythrocyte culture. Samples without amplification (no Ct detected) were considered as negative, and a density of 2 parasites/μL was assigned if amplification was observed out of the lower range of the standard curve (5 parasites/μL). Purified DNA from the 3D7 parasite strain was used as a positive control, whereas a negative control with no DNA template was run in all reactions. *Plasmodium falciparum* infections detected by qPCR, but not by microscopy, were classified as submicroscopic.

### Molecular genotyping of the Polymorphic Genes *msp-2* and *glurp*

In this study, a fragment-analysis method was used to enumerate all of the *P. falciparum* genotypes and to quantify their proportions within isolates [14]. This method is based on the polymorphism of the gene encoding for merozoite surface protein 2 (MSP-2), an abundant surface component in the erythrocyte-invading stage of *P. falciparum* [15]. A highly polymorphic region showing considerable variations in size and sequence has been described in block 3 of the *msp-2* gene [16]. Women’s samples showing the same *msp-2* profile on both before-pregnancy and early-pregnancy samples were then analyzed on the *glurp* (glutamate-rich protein) marker, which is also a highly polymorphic marker in *P. falciparum* [17, 18]. Thus, by using 2 highly polymorphic markers, we aimed to obtain better genotype identification when comparing pairs of isolates for each woman [17, 19, 20].

Analyses of a fluorescent qPCR block 3 of the *msp-2* and *glurp* domains were conducted as described in a previous study [14]. This method is based on a qPCR amplification with a fluorescent primer, followed by capillary gel electrophoresis, processed in an ABI Prism 3130 XL Genetic Analyzer (Perkin Elmer Applied Biosystems) to enumerate and quantify fluorescent fragments, and therefore to discriminate alleles of different sizes in each isolate. The fragment-analysis method was processed in the sequencing platform at Bichat Hospital (Paris, France). The true value of this method has already been evaluated in clinical settings in previous studies [19–22]. This method is simple to use, rapid, and practical. Our ability to differentiate new infections from persistent infections in in vivo studies may be more precise in comparison to other methods because the proportion of each genotype (previous and new) is determined in a polyclonal isolate. This amplification was based on the semi-nested PCR technique reported by Snounou et al [23]. Data obtained from the ABI Prism 3130 XL Genetic Analyzer were analyzed using GeneMapper software (version 8; Applied Biosystems). Each genotype was characterized by the size and the area under the curve of the peak corresponding to the *msp-2* and *glurp* qPCR products.

### Statistical Analysis

First, all the samples were genotyped for the *msp-2* polymorphic marker. We distinguished 2 different groups of women: (1) women with the same infection profile before and in early pregnancy, and (2) women with different infections. We considered 2 infections as identical in the following cases: (1) with the same number of genotypes and identical genotypes (identical infection profile), and (2) with 1 or 2 minority genotypes (<2%) that appeared or disappeared between the 2 time points in each woman. All women who had an identical *msp-2* profile infection on both consecutive samples were analyzed for the *glurp* RII region marker. An infection was considered as persistent when identical *msp-2* and *glurp* genotypes were found in the corresponding prepregnancy and early-pregnancy samples. In this case, the 2 profiles were either completely identical for the same marker or they had 1 or 2 missing or additional minority genotypes.

Baseline and malaria characteristics at inclusion before conception and during pregnancy are presented for women included in RECIPAL, and those included in the present genotyping study. Based on both *msp-2* and *glurp*, the number (proportion) of women with an identical infection before and in early pregnancy was calculated and was then classified according to the type (microscopic vs submicroscopic) of infection before pregnancy.

Stata version 13 for Windows (StataCorp) was used for all statistical analyses.

## RESULTS

Among the 66 women who met the inclusion criteria (positive qPCR for isolates from both before pregnancy and early pregnancy), 3 had no results for *msp-2* genotyping (missing data).

The main characteristics of the women included in the RECIPAL project and of the 63 of those who were included in the genotyping study are presented in Table 1. Most women included in the present study were multigravid (89%). Among the 63 women in the current study, only 11 (17.5%) presented with a microscopic infection before pregnancy. The median time between the 2 malaria screenings was 96 days (interquartile range [IQR], 42–193 days). The median gestational age at the first ANC visit was 6.4 weeks (IQR, 5.1–7.1 weeks).

**Table 1.** General and Malaria Characteristics of Women Whether or Not Included in the Genotyping Analysis: Benin, 2014–2017

Characteristics	Women Included in the Genotyping Study (n = 63/411)	Women Not Included in the Genotyping Study (n = 348/411)
General characteristics		
Mean ± SD age, years	26.2 ± 5.3	26.9 ± 5.0
Primigravid, n (%)	7 (11.1)	26 (7.5)
Illiterate, n (%)	50 (79.4)	240 (69.0)
HIV positive, n/N (%)	2/56 (3.6)	4/298 (1.3)
Low socioeconomic level,^a^ n (%)	25 (39.7)	115 (33.1)
At inclusion before conception		
Anemia,^b^ n (%)	37 (58.7)	184 (53.3)
qPCR-positive infection, n (%)	63 (100)	53/320 (16.6)
Parasite density,^c^ parasites/µL	46 (25–82)	15 (8–27)
Microscopic infection, n (%)	11 (17.5)	12/320 (3.8)
Submicroscopic infection,^d^ n (%)	52 (82.5)	47/53 (88.7)
During pregnancy		
Median (IQR) time between malaria screening before/in early pregnancy, days	96 (42–193)	125 (57–233)
At the first ANC visit		
Median (IQR) gestational age at the first ANC visit, weeks	6.4 (5.1–7.1)	6.4 (5.4–7.7)
qPCR-positive infection, n (%)	63 (100)	68/317 (21.5)
Parasite density,^c^ parasites/µL	46 (23–91)	38 (19–79)
Microscopic infection, n (%)	9 (14.3)	20/317 (6.3)
Submicroscopic infection,^d^ n (%)	54 (85.7)	58/68 (85.3)
During the entire pregnancy, n (%)		
At least 1 microscopic infection^e^	34 (54.0)	106 (30.5)
Placental malaria infection	4/36 (11.1)	11/199 (5.5)

Abbreviations: ANC, antenatal care; HIV, human immunodeficiency virus; IQR, interquartile range; qPCR, quantitative polymerase chain reaction; RECIPAL, REtard de Croissance Intra-utérin et PALudisme

^a^Socioeconomic level was approximated using a synthetic score combining occupation and ownership of assets, which was then categorized according to tertiles within the whole RECIPAL cohort; a “low” socioeconomic level corresponds to the lower tertile.

^b^Anemia before conception defined as a hemoglobin level <12 g/dL.

^c^Values are geometric means (95% confidence interval); parasite density by qPCR was calculated in positive qPCR specimens.

^d^Proportion of submicroscopic infection (ie, qPCR-positive but thick blood smear test–negative) among qPCR-positive infections; overall proportion of submicroscopic infections before conception (47/320, 15.3%) and at the first ANC visit (58/317, 19.5%).

^e^At least 1 microscopic infection during pregnancy or at delivery.

**Figure 1.**
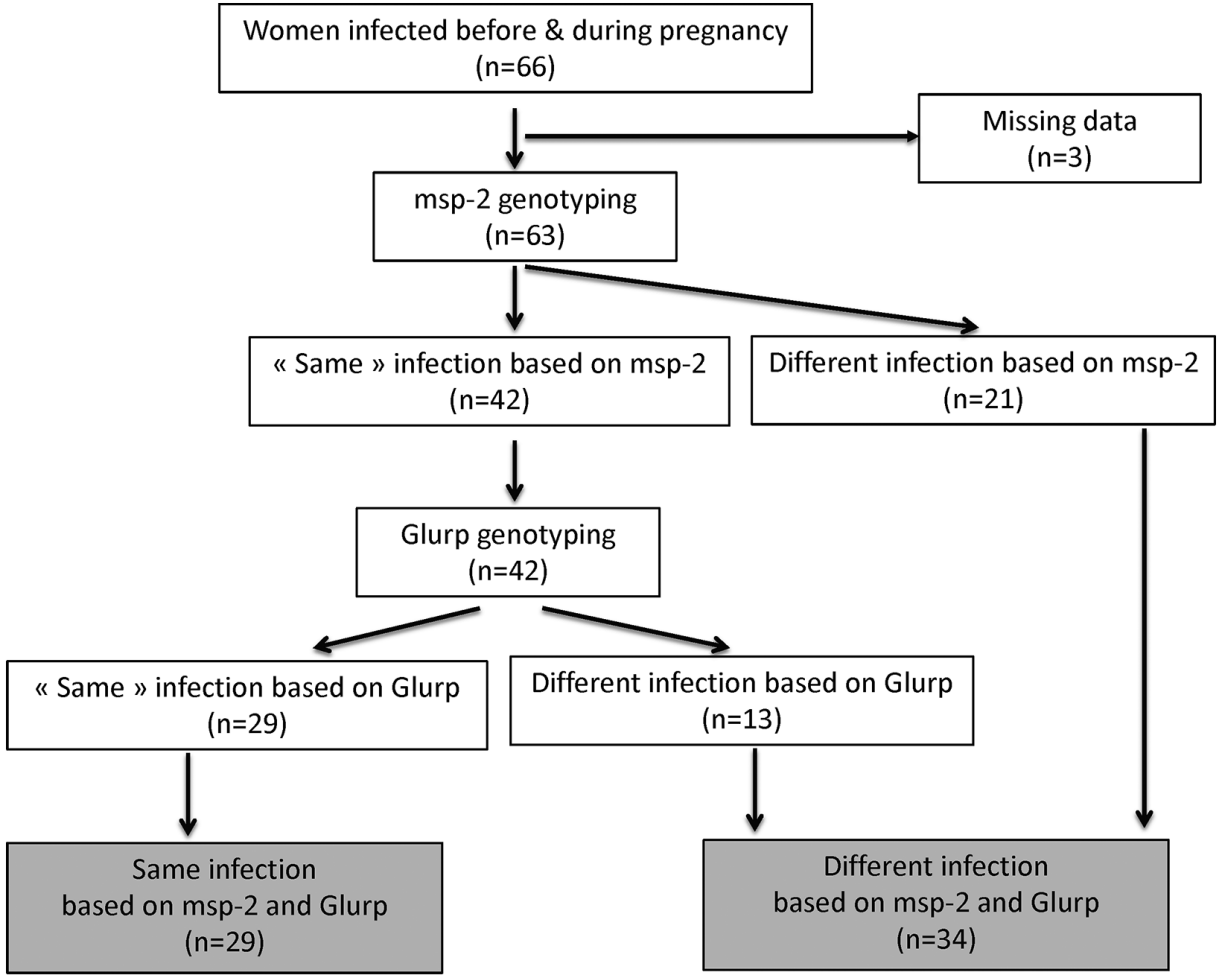
Results of *msp-2* (merozoïte surface protein-2) and *glurp* (glutamate-rich protein) genotyping: Benin, 2014–2017.

Results of *msp-2* and *glurp* genotyping are presented in Figure 1. Based on only *msp-2* genotyping, 42 of 63 women (66.7%) had the same infection profile before pregnancy and in early pregnancy. Based on both *msp-2* and *glurp* genotyping, the infection was considered similar before and in early pregnancy in 46% (29/63) of cases (Figure 1).

**Figure 2.**
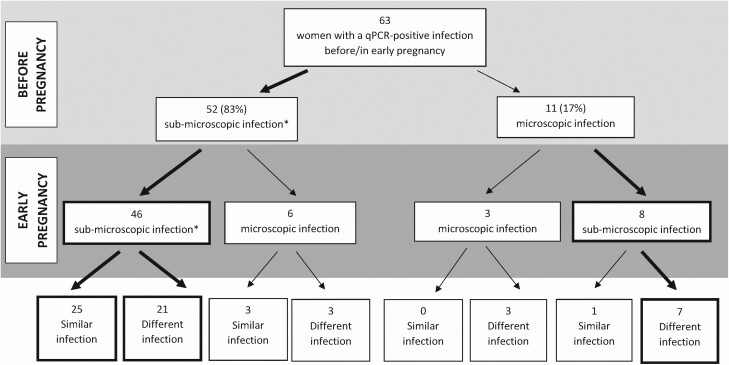
Profiles of infection (submicroscopic/microscopic and similar/different infection) before pregnancy and in early pregnancy. RECIPAL: Benin, 2014–2017. Bold arrows indicate the most frequent profiles. *Submicroscopic infections were *Plasmodium falciparum* infections detected by qPCR but not by microscopy. Abbreviations: qPCR, quantitative polymerase chain reaction; RECIPAL, REtard de Croissance Intra-utérin et PALudisme.

Figure 2 presents different profiles of infection at 2 time points: before pregnancy and at the first ANC visit. Among the 63 included women, 52 (83.0%) had a submicroscopic infection before pregnancy. Most of them (46/52; 88.5%) still had a submicroscopic infection in early pregnancy, with an infection profile similar to that before pregnancy for more than half of the women (25/46; 54.3%). The median time between the 2 malaria screenings was higher for the 21 women with a different infection profile (4 months) compared with the 25 women with a similar infection profile (3 months) before and in early pregnancy. Eleven women (17%) had a microscopic infection before pregnancy. The majority (7/11) presented with a submicroscopic infection with a different profile of infection in early pregnancy. Presenting with a microscopic malaria infection in early pregnancy was more likely in women with a microscopic infection before pregnancy (3 out of 11 women) compared with women with a submicroscopic infection before pregnancy (6 out of 52 women).

## Discussion

Our findings show the persistence of malaria infection from the preconceptional to the early-pregnancy period. We showed that a high proportion (46%) of PCR-positive infections detected at the beginning of the first trimester of pregnancy were already present before pregnancy. Most of these infections were submicroscopic as they were only detected by qPCR.

Following World Health Organization recommendations, insecticide-treated mosquito nets and IPTp-SP are currently used in all areas in Africa with moderate to high malaria transmission. However, the IPTp-SP treatment excludes the first trimester of pregnancy, a time frame when the infections are frequent and harmful to pregnant woman and their fetus [5, 24–26]. Previous epidemiological studies have shown a high prevalence of malaria infection often observed at the first ANC visit in pregnant women who did not receive IPTp-SP [27–29]. One explanation is the low use of bed nets by young women in poor areas [30, 31]. Moreover, a high prevalence of asymptomatic (thus untreated) infections in adolescents and young women has been reported in several studies [7]. Another factor that may potentially contribute to the high prevalence of malaria in early pregnancy is the selection of a pregnancy-specific parasite phenotype, specifically expressing the var2csa member of the var multigene family [32, 33]. Our previous findings suggest that the variant var2csa represents a minor population in parasite isolates before pregnancy, which can be rapidly selected in vivo under favorable physiological conditions [8, 34]. Complementary phenotyping analyses are ongoing to assess whether those pregnancy-specific parasites in early pregnancy originate from a specific population of parasites before conception (T. Ndam, personal communication, 2020).

Berry et al [35] previously assessed the hypothesis of malaria infections persisting into pregnancy. They showed that as many as 41% of women at the first ANC visit were infected with malaria when their entire pregnancy before the first ANC had occurred in the dry season. Another recent study based on the level of VAR2CSA-specific immunoglobulin G (IgG) among Ghanaian women at their first ANC visit reinforces the notion that placental malaria often arises from parasites already present at the time of conception [36]. Finally, in Benin, genotyping of recurrent infections from 18 primigravid women infected before pregnancy led to results comparable to ours, namely a high proportion of infections were persistent from the preconceptional period [8]. The present study completes the first one from Benin by including multigravid women and by using a more sensitive genotyping approach. However, these results may only be generalizable to epidemiological contexts similar to that of South Benin.

The strength of this study is the use of 2 highly polymorphic markers, *msp-2* and *glurp*, which provide a higher sensitivity in the detection of *P. falciparum* genotypes. *Msp-1*, circumsporozoite protein (*csp*), and other parasite polymorphic markers usually used in genotyping studies are less discriminant. Based on both *msp-2* and *glurp* genotyping, the percentage of women with a persistent infection dropped from 66% (only *msp-2*) to 46% (*msp-2* and *glurp*). We assume that the use of *glurp* as a highly polymorphic marker is an added value since it makes this genotyping method more informative. We used a fragment-analysis method to enumerate the *P. falciparum* genotypes and to quantify their proportions within the isolates. The area under the curve is proportional to the quantity of PCR amplicons for each genotype [37]. Our data show that genotypes with a minor frequency in the isolate disappear most of the time or persist with the same ratio in the isolate. Furthermore, this method detects minority genotypes compared with classical molecular methods. This approach consistently identified genotypes as low as 0.5%. Comparable sensitivity has been obtained in studies using 32P-labeled primers or probes, which are time-consuming techniques.

In our study population, the proportion of submicroscopic infections compared with microscopic infections was identical in the preconceptional and very-early-pregnancy period (82.5%). We therefore did not show that these subpatent infections in the preconceptional period become microscopically detectable in early pregnancy. This was recently demonstrated by Tuike Ndam et al [8] in Benin, although in their study, they considered all malarial infections until the fourth month of pregnancy. Previous results based on the RECIPAL study also showed that the ratio between submicroscopic and microscopic infection started to decrease at the end of the first trimester [7]. Our study design is therefore not suitable for demonstrating such a switch towards microscopic infections since malaria was detected very early in pregnancy (at ~6 weeks’ gestation) when the development of the placenta is not complete; no sequestration and multiplication of parasites are therefore possible in such an environment. Another explanation of the high prevalence of submicroscopic infections in early pregnancy may be the high percentage of multigravid women in our selected study group (83%). The development of a specific immunity against the parasite during previous pregnancies must be taken into consideration in this case [3].

In addition, the fact that our study population mainly included multigravida women may partly explain the apparently low proportion of pregnant women with persistent infection (29/411). Other factors probably contributed to this. Indeed, 11 women out of 63 (17%) had a microscopic infection before pregnancy for which they were treated with artemether-lumefantrine. In addition, women infected both before and during pregnancy were identified based on a single screening before pregnancy. Finally, the elapsed time of 3 months between the 2 screenings possibly increased the risk of de novo infection. Therefore, the overall proportion of women with a persistent infection may be higher than that reported in the RECIPAL framework.

There is growing evidence that, in the first trimester of pregnancy, *Plasmodium* infection greatly contributes to the burden of malaria in pregnancy and has major deleterious clinical consequences on maternal and child health [5, 24–26]. Therefore, clearance of infection or prevention of infection during this period is likely to have a major effect both on the number of women with placental infections and on the deleterious effects of these infections [9]. In early pregnancy, only insecticide-treated bed nets are recommended for the prevention of malaria, but the coverage is very low; IPTp-SP is only recommended after the first trimester of pregnancy because of potential teratogenic effects of SP. Currently, there are no other appropriate and available measures that can be used in this critical period of pregnancy, with the knowledge that reaching women during this period is challenging since women in Africa usually do not attend the maternity clinic before 4 or 5 months of pregnancy.

Our data confirm that a high proportion of *P. falciparum* infections detected in the first trimester result from the persistence of the infection acquired before pregnancy. Therefore, we believe that targeting young women living in endemic areas protects them from malaria infection before their pregnancy, and might therefore reduce the prevalence of malaria in the first trimester of pregnancy, and its related poor maternal and birth outcomes. Such strategies may rely on highly sensitive diagnostic tests that help in the identification of women who need to be treated before pregnancy.

## Notes

*Acknowledgments.* **The authors are extremely grateful to all women who took part in this study; the midwives, nurses, and community health workers for recruiting and following them; and the whole RECIPAL team, including engineers and technicians, who were key in obtaining high-quality data and samples.**

*Financial support.* **This work was supported by the Bill and Melinda Gates Foundation (grant number OPP1169555). The RECIPAL project was supported by the French Agence Nationale de la Recherche (grant number ANR-13-JSV1-0004, 2013)** **and the Fondation Simone Beer under the auspices of the Fondation de France (grant number 00074147, 2017).**

*Potential conflicts of interest.* **The authors: No reported conflicts of interest. All authors have submitted the ICMJE Form for Disclosure of Potential Conflicts of Interest.**
